# Combined Medial Patellofemoral Ligament and Medial Quadriceps Tendon-Femoral Ligament Reconstruction With Single Knotless Anchor

**DOI:** 10.1016/j.eats.2024.103102

**Published:** 2024-06-29

**Authors:** Mathew Hargreaves, Hamp Sisson, Chandler Harris, Amit M. Momaya, Aaron J. Casp

**Affiliations:** a Department of Orthopaedic Surgery, University of Alabama at Birmingham, Birmingham, Alabama, U.S.A.; b Heersink School of Medicine, University of Alabama at Birmingham, Birmingham, Alabama, U.S.A.

## Abstract

Lateral patellar dislocations represent a common orthopaedic condition in young, active individuals who experience patellar instability. Despite increasing interest in re-creating the natural biomechanics and native knee anatomy when performing medial patellar soft-tissue reconstructive procedures, there is no consensus among surgeons regarding the best techniques to use when reconstructing the medial patellofemoral ligament and medial quadriceps tendon-femoral ligament. This article details a method to reconstruct the medial patellofemoral ligament and medial quadriceps tendon-femoral ligament using a single graft and a single all-suture knotless anchor on the patella.

Patellar dislocations are a common orthopaedic injury, with an annual incidence of 23.2 patellar dislocations per 100,000 person-years.^1^ Dislocations can then develop into chronic lateral patellar instability. This condition is influenced by several biomechanical and anatomic factors including generalized hyperlaxity, chronic ligamentous laxity, bony malalignment, trochlear dysplasia, connective tissue disorder, and anatomic pathology.^2^ The medial patellofemoral ligament (MPFL) is considered the primary restraint to lateral patellar displacement, preventing dislocation.^3^^,^^4^

The MPFL originates between the adductor tubercle and medial epicondyle of the femur and attaches to the medial aspect of the patella.^4^ The medial quadriceps tendon-femoral ligament (MQTFL) is an extension of the MPFL’s patellar attachment and acts as a stabilizing structure within the knee joint. These ligaments establish a stable connection between the medial femur and the anterior knee extensor mechanism during knee flexion-extension.^5^

Surgical MPFL reconstruction is a common treatment for recurrent instability, and many differences in surgical technique exist, with variations in graft selection, suture choice, and tunnel positioning.^6^, ^7^, ^8^ Many techniques currently used to address lateral patellar instability focus on reconstructing the MPFL or MQTFL in isolation,^3^^,^^5^ and the current literature lacks a consensus regarding the optimal technique for reconstructing the medial patellar ligament complex.^3^^,^^7^ Newer surgical procedures have combined multiple techniques to restore normal knee anatomy and biomechanics to address the medial complex as a whole.^9^, ^10^, ^11^ This article describes a combined MPFL and MQTFL technique using a single graft and a single all-suture knotless anchor on the patella.

## Surgical Technique

### Indications

Combined MPFL-MQTFL reconstruction with a single knotless anchor is indicated for patients experiencing recurrent patellofemoral instability. Video 1 demonstrates our surgical technique.

### Positioning and Examination

**Fig 1:**
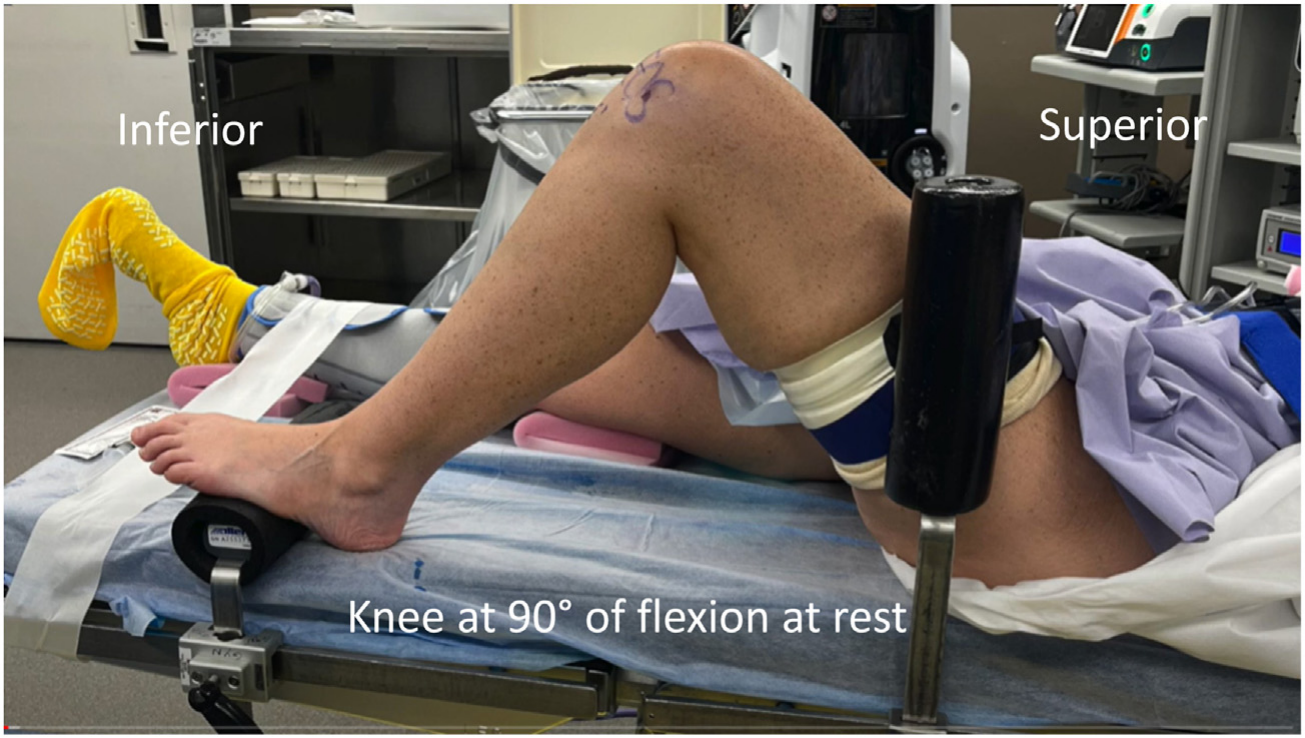
Patient is positioned supine with a non-sterile tourniquet around the left thigh. Lateral thigh and foot posts are positioned so that the knee can rest at 90 degrees of flexion.

The patient is positioned supine with a nonsterile tourniquet on the operative leg. After induction of anesthesia, an examination is performed to assess the degree of pathologic lateral patellar translation or any other concomitant ligamentous laxity. A lateral thigh post and foot post are attached to the bed so that the knee can rest at 90° of flexion (Fig 1). The limb is then prepared and draped in the standard sterile fashion.

### Graft Preparation

A non-irradiated semitendinosus allograft tendon at least 220 mm in length is whipstitched on either end with high-strength nonabsorbable suture, leaving the tails coming off the ends of the graft. The graft ends are tubularized during their suturing process so that both tails together can fit through a 7-mm-diameter sizer. The prepared graft is then allowed to soak in 1 g of vancomycin reconstituted in 500 mL of normal saline solution.

### Arthroscopy

Arthroscopic intervention is first performed through standard anterolateral and anteromedial arthroscopic knee portals. This allows us to visualize the trochlea and lateral patellar translation directly and address any intra-articular or chondral pathology. Attention is then turned to the MPFL and MQTFL reconstruction with allograft.

##### Dissection

**Fig 2:**
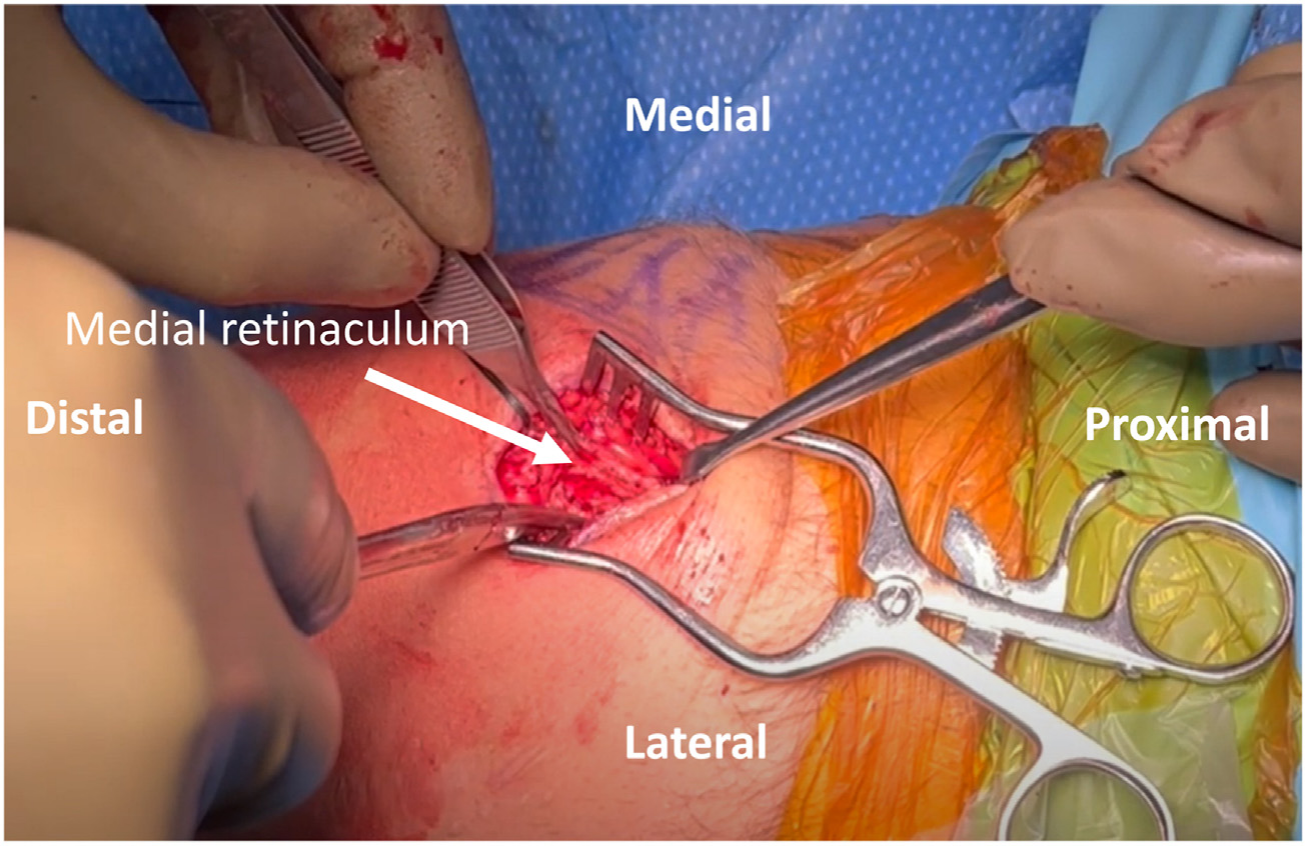
Patient is positioned supine with the left knee at 90 degrees of flexion. After careful dissection of the left knee, the medial retinaculum (arrow) is elevated off the medial aspect of the patella, and careful dissection is performed between layers 2 and 3 of the medial knee.

A 3-cm vertical incision is made on the superomedial border of the patella. Dissection is performed down to the anteromedial portion of the patella, creating a mobile window to expose the quadriceps tendon insertion on the patella. The medial retinaculum is elevated off the medial aspect of the patella, and careful dissection is performed between layers 2 and 3 of the medial knee, staying extra-articular and extending toward the medial femur (Fig 2). An incision is made along the medial thigh at the location of the femoral attachment of the MPFL. A nonabsorbable shuttling stitch is passed to connect the medial patellar incision with the medial femoral incision.

The medial patella is denuded of soft tissue, and a rongeur is used to make a bony trough on the medial edge of the superior half of the patella. This is the region where the graft will be incorporated to reconstruct the MPFL.

**Fig 3:**
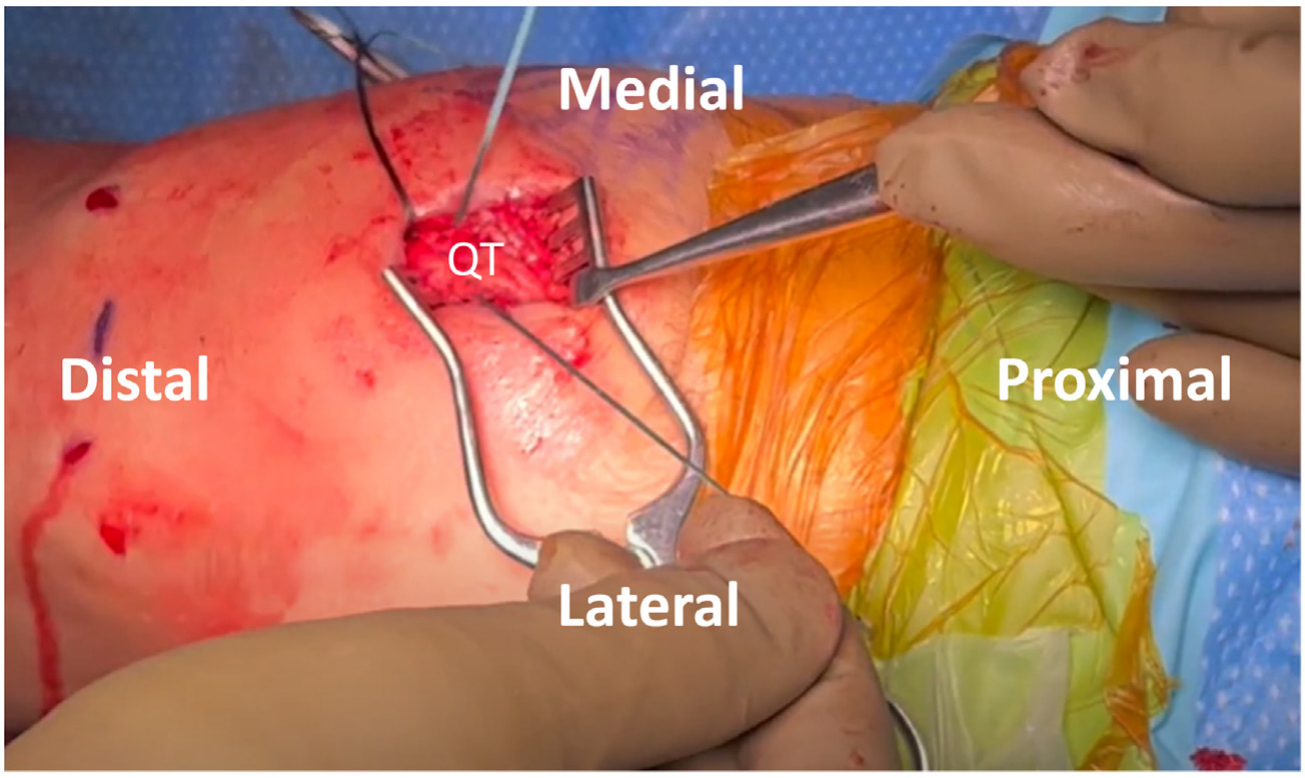
Patient is positioned supine with the left knee at 90 degrees of flexion. After a partial thickness tunnel is made through the quadriceps tendon, a passing suture is placed with the looped end exiting anterior.

For the MQTFL portion, a longitudinal partial-thickness slit is made in the distal quadriceps tendon, just proximal to the patellar attachment. This is tunneled to exit medially, superficial to the vastus intermedius contribution, to act as a partial-thickness tunnel through the quadriceps tendon. A passing suture is then placed with the looped end exiting anteriorly (Fig 3).

##### Reconstruction

**Fig 4:**
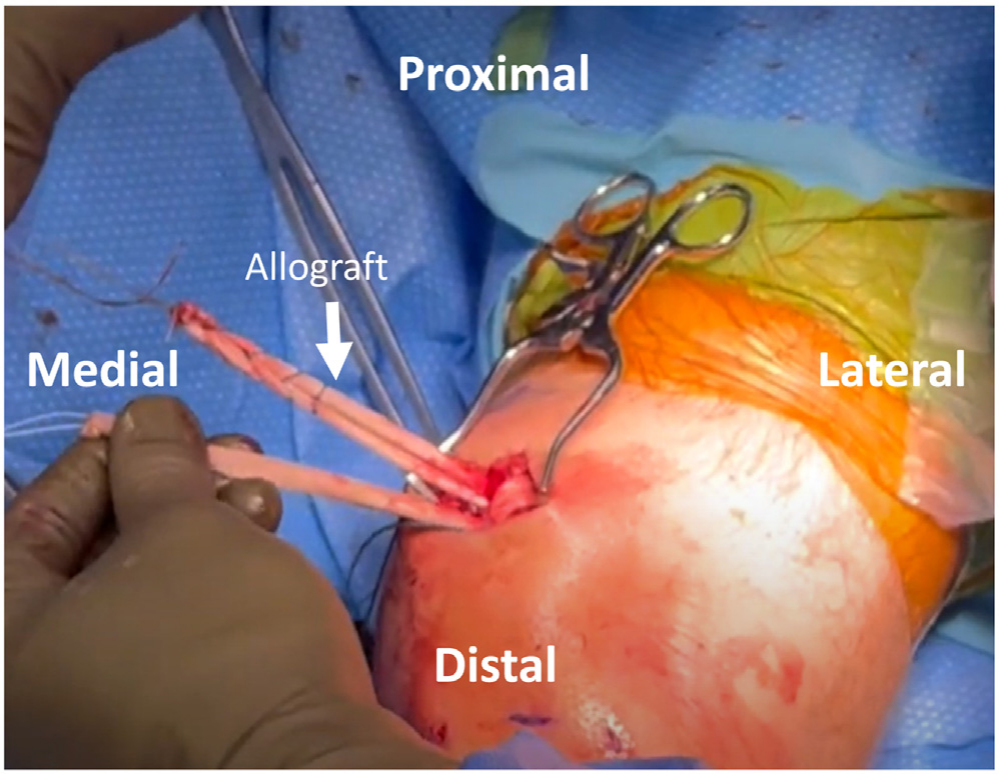
Patient is positioned supine with the left knee at 90 degrees of flexion. After the knotless, all-suture anchor is drilled and inserted into the medial patella, the proximal end of the allograft is passed anterior to posterior through the quadriceps tendon slit. The looped end of the graft is now attached to the patella and quadriceps tendon.

**Fig 5:**
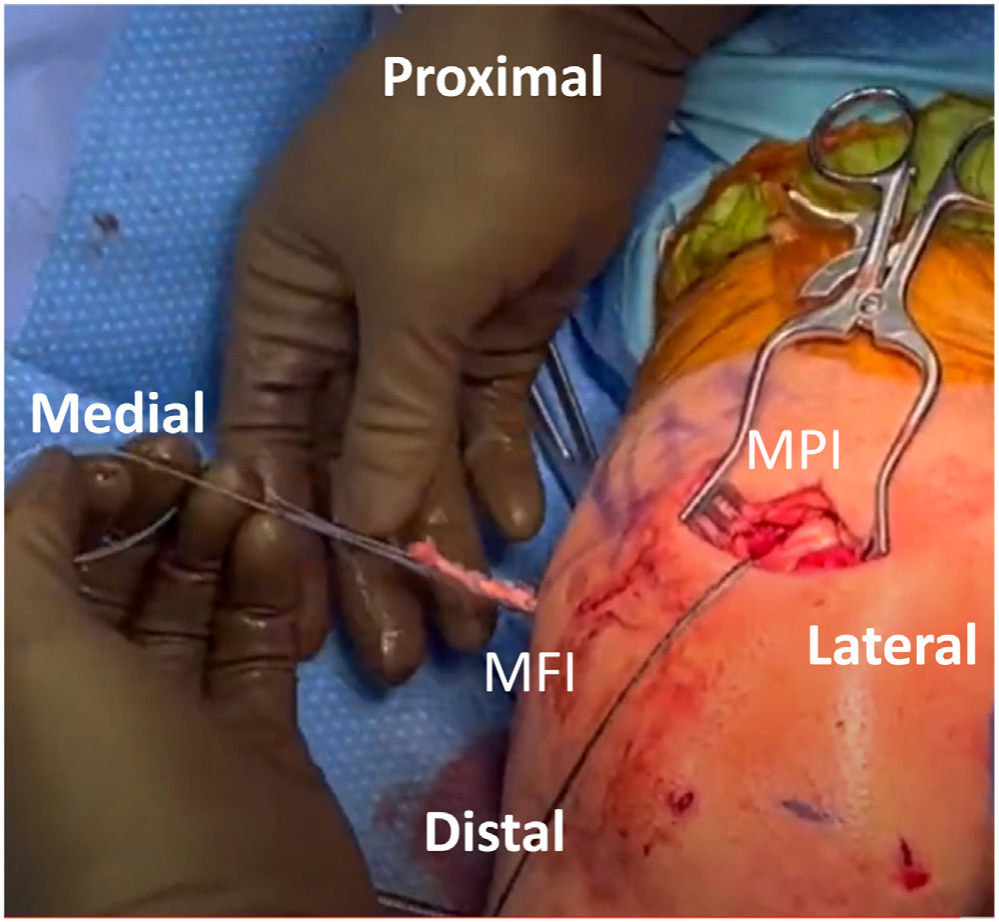
Patient is positioned supine with the left knee at 90 degrees of flexion. Using the previously placed shuttling stitch, both limbs of the allograft are shuttled from the medial patellar incision to the medial femoral incision.

An all-suture knotless anchor (Knee FiberTak; Arthrex) with double-looped tape is drilled and inserted into the medial patella within the previously created trough at the border of the upper one-fourth and lower three-fourths. The whipstitched allograft is then brought to the operative table and passed through the 2 loops of the knotless anchor on the patella. The proximal limb of the graft is adjusted to be approximately 2 cm longer to allow passage through the quadriceps tendon. The anchor is then cinched to reduce and compress the graft onto the prepared trough at the medial edge of the patella. The proximal end of the allograft is passed anterior to posterior through the quadriceps tendon slit using the previously placed passing stitch. The looped end of the graft has now been attached to the patella and the quadriceps tendon (Fig 4). At this point, both limbs of the allograft are roughly the same length and are shuttled from the medial patellar incision down to the medial femoral incision using the previously placed shuttling stitch (Fig 5).

**Fig 6:**
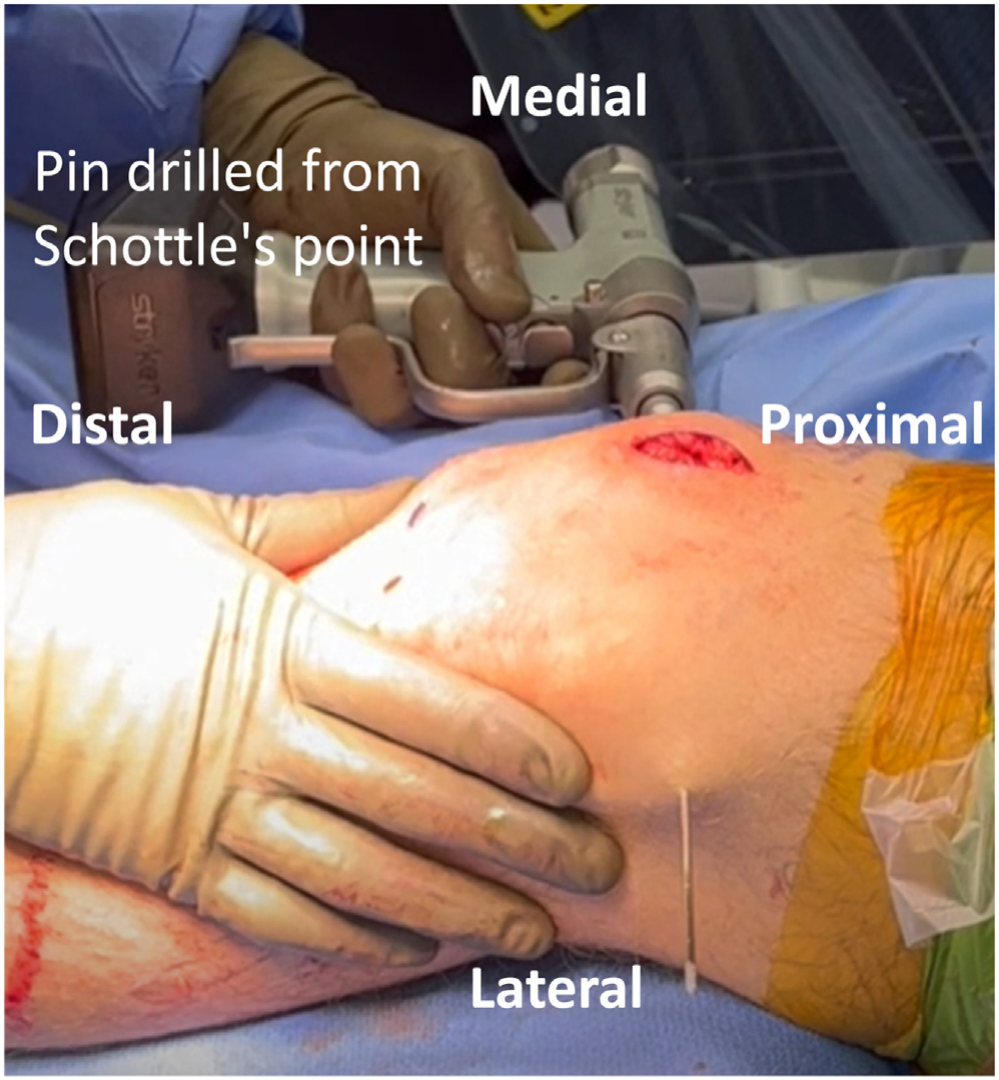
Patient is positioned supine with the left knee at 90 degrees of flexion. Starting at Schottle’s point (femoral attachment of the MPFL), a Beath pin is drilled from medial to lateral and exits the anterolateral.

By use of intraoperative fluoroscopy, the Schöttle point is localized as the femoral attachment of the MPFL. A Beath pin is drilled from medial to lateral, starting at the Schöttle point and exiting the anterolateral thigh (Fig 6). The Beath pin is over-drilled with a cannulated reamer corresponding to the diameter of the 2 ends of the graft measured together, which is found by using a graft sizer. The suture tails from the 2 ends of the graft are shuttled through. The sutures are marked to identify them separately for each graft tail, enabling individual tensioning. This can be accomplished by using different-colored sutures for each graft end or marking the suture tails from 1 end with a sterile marking pen.

**Fig 7:**
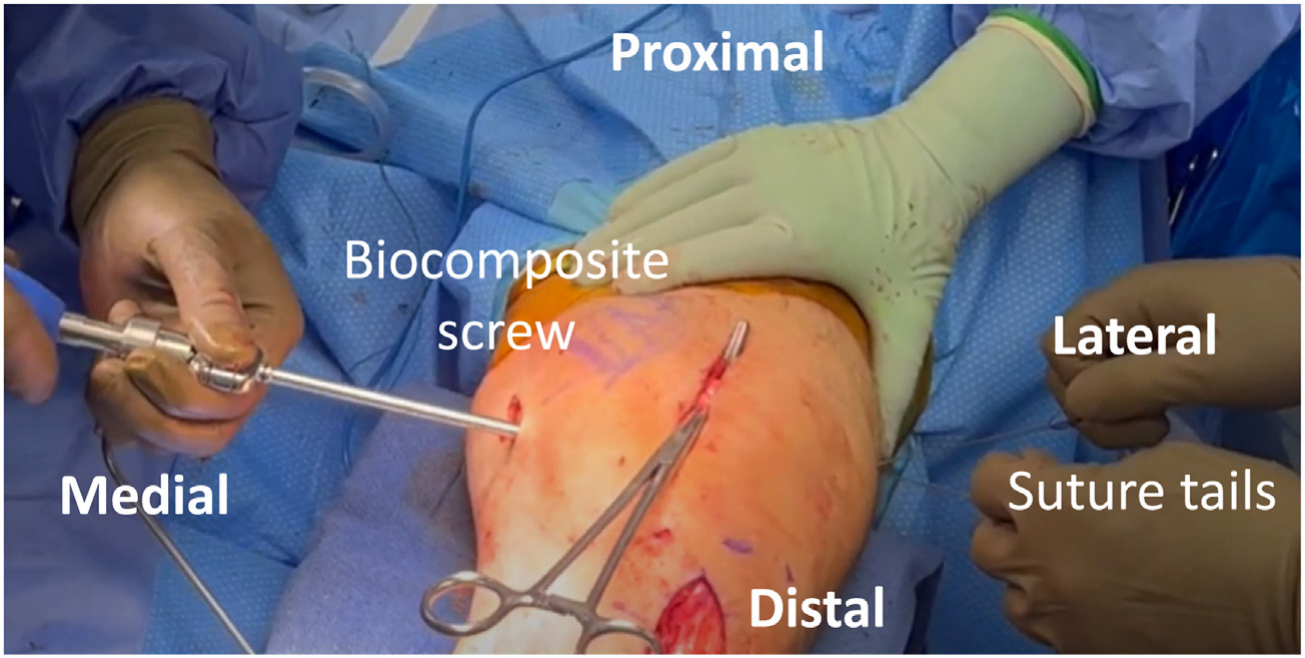
Patient is positioned supine with the left knee at 60 degrees of flexion. Tension is placed on the suture tails of the graft emerging from the lateral thigh. A hemostat is placed in the medial patellar incision underneath the graft to prevent over-tensioning. A biocomposite tenodesis screw (Arthrex, Inc) is placed in the medial femur tunnel to tension the graft tails.

The knee is placed at roughly 60° of flexion on a radiolucent triangle. Tension is placed on the suture tails of the graft emerging from the lateral side of the thigh. To prevent over-tensioning of the graft and overconstraint of the patellofemoral joint, a hemostat or Kelly clamp is placed in the medial patellar incision underneath the graft to provide laxity. The graft tails are tensioned, and a 7 × 23-mm BioComposite tenodesis screw (Arthrex) is placed into the medial femur tunnel, completing the reconstruction (Fig 7). Table 1 presents pearls and pitfalls of our technique, and Table 2 lists advantages and disadvantages.

**Table 1:** Pearls and Pitfalls

Pearls
Correct identification of the Schöttle point via fluoroscopy is necessary to find the femoral attachment of the MPFL.
The single all-suture knotless anchor allows for a smaller drill hole on the patella.
A proper understanding of the knee’s anatomy is necessary for graft passage.
Pitfalls
Over-tensioning the graft can result in patellofemoral stiffness and residual pain.
The inability to determine alternate patellar instability causes intraoperatively may result in continued instability postoperatively.
Improper tunnel and anchor placement can increase patellar fracture risk.
Surgeons should exercise care not to extend into the joint capsule while developing the intertendinous portion of the quadriceps dissection.

MPFL, medial patellofemoral ligament.

**Table 2:** Advantages and Disadvantages

Advantages
Restoration of normal knee anatomy and biomechanics
Increased patellofemoral stability
Smaller drill hole on patella and less patellar bone violation
Beneficial in patients with small patella owing to single anchor point rather than placement of 2 anchors placed
Reduction in costs associated with operation owing to use of 1 anchor
Disadvantages
Small risk of overconstraint with resulting patellofemoral stiffness
Increased incision size to address quadriceps tendon
Possibility of quadriceps inhibition due to quadriceps tendon incorporation

### Rehabilitation

After initial wound closure and dressing, the patient’s knee should be placed in a knee immobilizer. The patient may be allowed to use crutches and perform light to intermediate mobilization of the knee during the 6-week period after surgery. The return to normal daily activities should happen gradually, with support and guidance from a physical therapist. With appropriate therapy, a full return to sports may occur at 4 months.

## Discussion

This article illustrates a surgical technique for combined MPFL and MQTFL reconstruction that uses a single graft and a single all-suture knotless anchor on the patella with the aim of restoring the knee’s native anatomy. This technique builds on recent advances in surgical techniques in which additional anatomic knee structures are repaired alongside the MPFL.^9^ Restoration of the knee’s intact state through combined MPFL and MQTFL techniques results in a strong patellar constraint, which can lead to effective patient outcomes with reduced future dislocations.^10^^,^^11^ The optimal techniques for MPFL repair have been debated, and there is currently no consensus in the literature on the best technique to address chronic lateral patellar instability.^3^ Many techniques have focused on isolated MPFL repair, whereas others have addressed isolated MQTFL repair.^3^^,^^5^ The techniques focused on surgically fixing chronic lateral instability vary in graft selection, suture choice, and tunnel positioning, suggesting that there is a need for more discussion regarding the best surgical techniques for MPFL repair.^6^, ^7^, ^8^

Compared with previous techniques that reconstructed the MPFL or MQTFL in isolation, combined MPFL and MQTFL reconstruction anatomically reconstructs the medial knee structures that confer patellofemoral stability. The single all-suture knotless anchor allows for a smaller drill hole on the patella compared with a solid anchor and less patellar bone violation. This technique is particularly advantageous for patients with a small patella because the single anchor point is easier to place in a small patella compared with 2 anchors. Finally, the single anchor reduces the cost associated with the procedure.

However, there are some potential disadvantages to the described technique. There is a small risk of over-tensioning, which could result in patellofemoral stiffness. This technique requires a slightly larger incision to address the quadriceps tendon, which could increase infection risk and scar length. There is also potential for quadriceps inhibition due to incorporation into the quadriceps tendon. Finally, surgeons should be careful not to extend into the joint capsule while developing the intertendinous portion of the quadriceps dissection. With proper surgical technique and experience, the advantages of this procedure far outweigh the potential disadvantages.

In conclusion, this technical note describes combined MPFL and MQTFL reconstruction with a single all-suture knotless anchor for patients with persistent patellar instability. The combined MPFL and MQTFL approach allows for a more anatomic reconstruction of the medial knee structures than previous approaches described in the literature. The single all-suture knotless anchor reduces the cost of the procedure, decreases bony violation of the patella, and is ideal for patients with a small patella.

## Disclosures

The authors declare the following financial interests/personal relationships which may be considered as potential competing interests: A.M.M. reports a consulting or advisory relationship with 10.13039/100007307Arthrex, ConMed Linvatec, Fidia Pharma USA, and Miach Orthopaedics; reports board membership with *Arthroscopy*; and owns equity or stocks in Reparel. A.J.C. reports a consulting or advisory relationship with Arthrex. All other authors (M.H., H.S., C.H.) declare that they have no known competing financial interests or personal relationships that could have appeared to influence the work reported in this paper.

## Supplementary Data

Video 1 Combined medial patellofemoral ligament (MPFL) and medial quadriceps tendon-femoral ligament (MQTFL) reconstruction with single all-suture knotless anchor. The patient is positioned supine for the procedure, and this is the patient's left knee. The orientation of individual steps is denoted. The procedure aims to anatomically reconstruct both medial knee structures. The single all-suture knotless anchor reduces the cost of the procedure, decreases bony violation of the patella, and is appropriate for patients with a small patella. Specific technique steps are shown in greater detail.
